# How long to wait after local infiltration anaesthesia: systematic review

**DOI:** 10.1093/bjsopen/zrad089

**Published:** 2023-09-28

**Authors:** Mohammad Suleman Bajwa, Muhammad Mustehsan Bashir, Mohammad Hamza Bajwa, Zafar Iqbal, Muhammad Aizaz Salahuddin, Ahmad Hussain, Farooq Shahzad

**Affiliations:** Department of Plastic & Reconstructive Surgery/Mayo Burn Centre, Mayo Hospital, King Edward Medical University, Lahore, Pakistan; Department of Surgery, Montefiore Medical Center, New York, USA; Department of Plastic & Reconstructive Surgery/Mayo Burn Centre, Mayo Hospital, King Edward Medical University, Lahore, Pakistan; Department of Neurosurgery, Aga Khan University, Karachi, Pakistan; Department of Plastic & Reconstructive Surgery/Mayo Burn Centre, Mayo Hospital, King Edward Medical University, Lahore, Pakistan; Department of Plastic & Reconstructive Surgery/Mayo Burn Centre, Mayo Hospital, King Edward Medical University, Lahore, Pakistan; Department of Plastic & Reconstructive Surgery/Mayo Burn Centre, Mayo Hospital, King Edward Medical University, Lahore, Pakistan; Plastic & Reconstructive Surgery Service, Memorial Sloan-Kettering Cancer Center, New York, New York, USA

## Abstract

**Background:**

Conflicting evidence exists regarding the optimal waiting time for stable analgesic and vasoconstrictive effects after local infiltration of lidocaine with epinephrine. An objective review is needed to dispel surgical dogma.

**Methods:**

This systematic review (PROSPERO ID: CRD42022362414) included RCTs and prospective cohort studies. Primary outcomes were (1) onset of analgesia and (2) onset of stable hypoperfusion, assessed directly, or measured indirectly using perfusion imaging. Other data extracted include waiting strategies, means of outcome assessment, anaesthetic concentrations, volume/endpoint of infiltration, and injection sites. Methodological quality was evaluated using the Cochrane risk-of-bias tool for randomized trials. Articles describing waiting strategies were critically appraised by the Joanna Briggs Institute tools.

**Results:**

Twenty-four articles were analysed, comprising 1013 participants. Ten investigated analgesia onset. Their pooled mean was 2.1 min (range 0.4–9.0 min). This varied with anatomic site and targeted nerve diameter. Fourteen articles investigated onset of stable hypoperfusion. Four observed bleeding intraoperatively, finding the minimum time to hypoperfusion at 7.0 min in the eyelid skin and 25.0 min in the upper limb. The ten remaining studies used perfusion imaging, reporting a wide range of results (0.0–30.0 min) due to differences in anatomic sites and depth, resolution and artefacts. Studies using near-infrared reflectance spectroscopy and hyperspectral imaging correlated with clinical observations. Thirteen articles discussed waiting strategies, seven relating to large-volume tumescent local infiltration anaesthesia. Different waiting strategies exist for emergency, arthroscopic and cosmetic surgeries, according to the degree of hypoperfusion required. In tumescent liposuction, waiting 10.0–60.0 min is the norm.

**Conclusion:**

Current literature suggests that around 2 min are required for most patients to achieve complete analgesia in all sites and with all anaesthesia concentrations. Waiting around 7 min in eyelids and at least 25 min in other regions results in optimal hypoperfusion. The strategies discussed inform decisions of when and how long to wait.

## Teaser

The authors reconcile conflicting evidence regarding the optimal waiting time for stable analgesic and vasoconstrictive effects after local infiltration of lidocaine with epinephrine. Around 2 min are required for most patients to achieve complete analgesia in all sites and anaesthesia concentrations. Waiting around 7 min in eyelids at least 25 min in other regions results in optimal hypoperfusion. Strategies are discussed to inform the decisions of when and how best to wait.

## Introduction

Local infiltration anaesthesia (LIA) is widely used in surgery, dentistry and procedural specialties for analgesia of the surgical field. Epinephrine is frequently included in LIA for tissue vasoconstriction to reduce bleeding. These techniques improve patient safety and surgical efficiency^1–3^. Intraoperative patient participation may enhance functional outcomes; for example, the strength of tendon repairs can be gauged, and motor acquisition is faster^4^. Using LIA can reduce the need for narcotics and transfusions^2,5–9^.

Lidocaine is the most-used local anaesthetic. It blocks voltage-gated sodium channels in the cell membranes of sensory and vasomotor nerves^10^. It provides differential anaesthesia, affecting pain sensation more than fine touch and pressure^11–13^. Its onset of action is <2 min, with a duration of effectiveness of 1–2 h and a maximum dosage of 5 mg/kg^14^. The addition of epinephrine increases the duration of action to 2–6 h and increases the maximum allowable dosage to 7 mg/kg.

There is no clear consensus on the optimum time to wait after injecting LIA for stable analgesic and vasoconstrictive effects. The authors conducted a systematic review of the literature to evaluate latency periods of lidocaine and epinephrine in LIA. Factors influencing latency and relevant strategies and perspectives of different surgeons were also reviewed.

## Methodology

### Search and selection

This systematic review was conducted after protocol registration (PROSPERO CRD42022362414) and reported following the PRISMA statement^15^. Included were all randomized controlled trials and prospective cohort studies investigating the onset of analgesia and/or hypoperfusion after cutaneous injection of lidocaine with epinephrine. Excluded were animal studies, review articles and articles with incomplete descriptions of time, composition or concentration of LIA used. Articles on large nerve blocks and those limited to dental or mucosal LIA were excluded. No restrictions regarding year of publication or language of the article were applied. Studies of non-healthy human subjects were also included. The databases of PubMed (MEDLINE), Scopus and Google Scholar were systematically retrieved. The following keywords were used: ‘local anesthesia’, ‘local infiltration anesthesia’, ‘walant’, ‘walatt’, ‘time’, ‘waiting’, ‘latency’ and ‘onset’, with the Boolean operators ‘AND’, ‘OR’, and their combinations. The search included registries of clinical trials. Additional articles were found through secondary hand searches and through a citation-networking software (ResearchRabbit, Version 2.0, Human Intelligence Technologies, Incorporated). The search was last updated in April 2023.

### Data extraction and bias assessment

The titles and abstracts of the articles were reviewed independently by two reviewers. The full texts of articles that either reviewer found relevant were acquired. Full texts were assessed independently by two reviewers for relevancy. Disagreements were settled by discussion and by consultation with a third reviewer. In cases of missing information, an attempt was made to contact authors for details. Primary outcomes were (1) onset of analgesia and (2) onset of stable hypoperfusion, assessed directly or indirectly using perfusion-imaging techniques. Other data extracted include participant characteristics, waiting strategies, means of outcome assessment, LIA concentrations, volume/endpoint of infiltration, anatomic site of injection and calibrations of imaging instruments. The methodological quality of the included studies was independently evaluated by two reviewers using the Cochrane risk-of-bias tool for randomized trials (RoB2), based on the Cochrane Handbook for Systematic Reviews of Interventions, version 6.3^16^. The following item scales were assessed: random sequence generation ‘selection bias’, allocation concealment ‘selection bias’, blinding of the participants and personnel ‘performance bias’, blinding of outcome assessments ‘detection bias' and selective reporting ‘reporting bias’. Each item was graded as ‘Yes’ (low risk of bias), ‘No’ (high risk of bias) or ‘Unclear’ (unclear risk of bias). The risk-of-bias summary table was obtained using the robvis (risk of bias visualization) tool^17^. Studies’ conflicts of interest and sources of funding were also recorded.

For evaluating the articles containing expert opinions on waiting strategies, Joanna Briggs Institute tools were used^18^. Thus, only articles from experts that detailed logical and relevant strategies, considered extant literature and provided reasonable justifications were included. Unoriginal opinions and repetitions were not reported. Waiting strategies were stratified according to the volume of tumescent solution used, and substratified according to the type of surgery.

### Data synthesis

Studies were organized into groups: those describing onset of analgesia, and those describing onset of hypoperfusion. The second group was subdivided into those where hypoperfusion was determined by direct observation and those by non-invasive observation (by perfusion-imaging techniques). When depth and wavelength calibrations of the imaging machines used were not mentioned, commonly used calibrations were reported. Where possible, continuous data were analysed as pooled means with range, and dose–effect correlations were reported, using SPSS 23 (IBM Corp. released 2015, IBM SPSS statistics for Windows, version 23.0, Armonk, NY: IBM Corp.). In studies with multiple means of outcome assessment, the outcome with the latest mean time was used in pooled analysis. Outcomes of lidocaine-latency studies that included patients who received sedative premedications were excluded from the pooled mean analysis. Separate timelines were prepared to visualize lidocaine and epinephrine latency times across different studies.

## Results

### Results of search

**Fig. 1:**
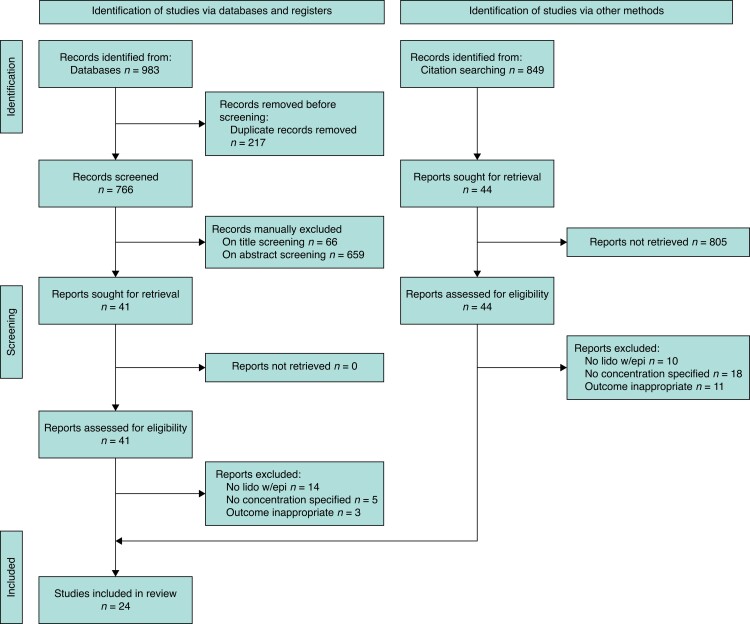
Preferred reporting items for systematic reviews and meta-analyses flowchart

The screening process and selection is depicted in *Fig. 1*. The preliminary search yielded 983 articles, reviews and letters, of which 766 were unique. After reviewing abstracts, 85 titles were selected for full-text screening. Twenty-four randomized controlled trials and cohort studies met eligibility criteria.

Thirteen articles on waiting strategies met selection criteria. Of these, six related to small-volume LIA and seven related to large-volume tumescent LIA.

### Included studies

**Fig. 2:**
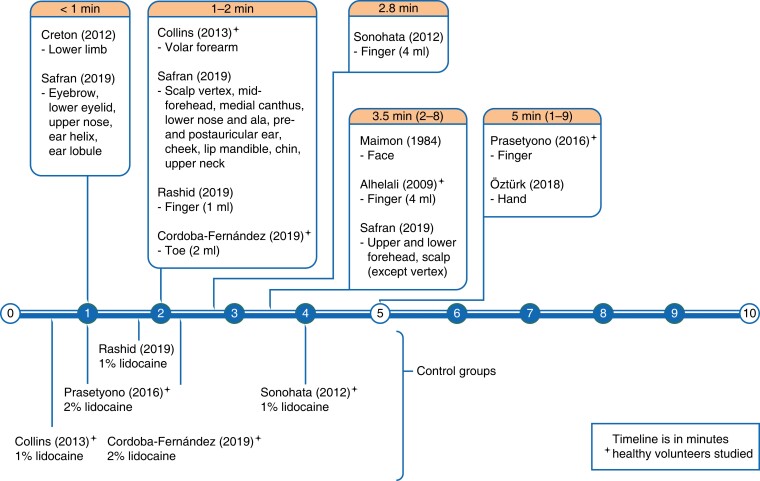
Timeline of studies on lidocaine latency/onset of analgesia

**Fig. 3:**
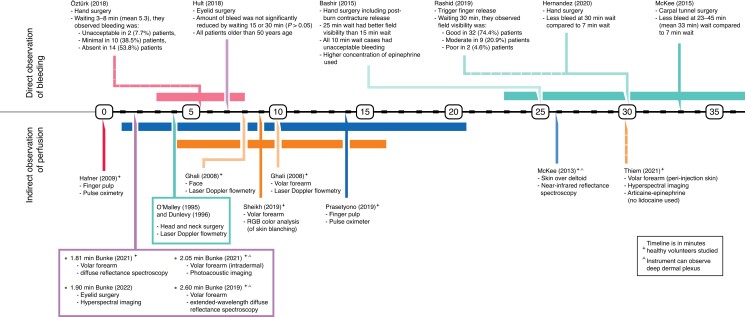
Timeline of studies on epinephrine latency/onset of hypoperfusion

A total of 24 articles were analysed, comprising 1013 participants, with ages ranging between 16 and 85 years^13,14,19–40^. Articles’ risk of bias is summarized in *Table 1*. The composite mean lidocaine and epinephrine concentrations were 7.05 (0.28–20.00) mg/dl, and 5.37 (0.14–20.00) µg/dl, respectively. The volume of LIA used ranged from 0.5 to 750.0 ml. There were 10 articles describing onset of analgesia with 628 total participants, 385 of whom received lidocaine with epinephrine LIA (see *Table S1*)^13,14,19–26^. Fourteen articles on the onset of hypoperfusion collectively had 335 participants. Of these, four articles observed patients’ intraoperative bleeding (*Table S2*)^27–30^, and 10 articles measured perfusion non-invasively (*Table S3*)^31–40^. Extracted data are organized in timelines (see *Fig. 2* and *Fig. 3*).

**Table 1:** Risk-of-bias visualization

Study	Random sequence generation (for selection bias)	Allocation concealment (for selection bias)	Blinding of participants and personnel (for performance bias)	Blinding of outcome assessment (for detection bias)	Complete outcome data (for attrition bias)	No selective reporting (for reporting bias)	Observation time ≥ 20 min	Buffer or additives beyond lidocaine and epinephrine (for confounder effect)	Healthy population studied (for Berkson’s bias)
**Onset of analgesic effect**									
Maimon *et al.*^13^	+	+	+	–	+	+	+	+	–
Alhelali *et al.*^20^	+	+	+	–	+	+	–	+	+
Sonohata *et al.*^21^	–	–	–	–	+	+	+	+	+
Creton *et al.*^19^	–	?	–	–	+	+	–	–	–
Collins *et al.*^14^	+	+	+	–	+	+	+	+	+
Prasetyono *et al.*^22^	+	+	+	–	+	+	–	+	+
Öztürk *et al.*^23^	?	?	?	–	+	+	+	–	–
Rashid *et al.*^24^	+	?	–	–	+	+	+	+	+
Safran *et al.*^25^	–	?	–	–	+	+	?	+	–
Córdoba-F. *et al.*^26^	+	+	+	–	+	+	+	+	+
**Onset of hypoperfusion—directly observed**									
Bashir *et al.*^27^	+	+	+	–	?	+	+	+	–
McKee *et al.*^28^	–	–	–	–	+	+	–	+	–
Hult *et al.*^29^	+	+	+	–	–	+	+	+	–
Hernandez *et al.*^30^	–	–	–	–	+	+	+	+	–
**Onset of hypoperfusion—indirectly observed**									
O'Malley *et al.*^31^	?	–	+	–	+	+	–	+	–
Dunlevy *et al.*^32^	+	–	+	–	+	+	–	+	–
Ghali *et al.*^33^	+	?	+	–	+	+	+	–	+
Hafner *et al.*^34^	+	?	+	–	+	+	+	+	+
McKee *et al.*^35^	+	+	+	+	+	+	+	+	+
Sheikh *et al.*^36^	+	?	?	–	?	+	–	–	+
Prasetyono *et al.*^37^	+	+	+	–	+	+	+	+	+
Bunke *et al.*^38^	?	?	?	–	?	+	–	+	+
Bunke *et al.*^39^	+	?	?	–	+	+	–	+	+
Bunke *et al.*^40^	+	?	?	–	+	+	–	+	–

+, low risk of bias; –, high risk of bias; ?, unclear risk of bias.

### Latency of lidocaine

The pooled mean of the latency of lidocaine (with epinephrine) was 2.6 min (range 0.4–9.0 min). This is greater than the pooled mean of plain lidocaine latency (1.7 min). Of the 10 studies reviewed, pain was assessed by sharp prick in eight, and upon surgical incision in three studies. One study also assessed two-point discrimination. There were variations with anatomic site, with a trend towards longer waiting time in larger nerves and thicker skin regions (see *Fig. 2*). Collins *et al.* observed analgesia before 1 min in the volar forearm^14^. In head and neck skin cancer patients, Safran *et al.* observed rapid analgesia (≤1.0 min) with a smaller volume of LIA (≤3.0 ml) in the eyebrow, lower eyelid, upper nose, ear lobule and helix. They also found late analgesia (≥2.0 min) despite a large volume of LIA (≥6.0 ml) in the forehead, mid-scalp and occipital scalp^25^. Vertex scalp (3.0 mm thick) took 1.2 min, whereas occipital scalp (8.0-mm thick) took 3.5 min^25,41^. Digital analgesia using 4 ml of LIA took 2–8 min to achieve^20,21^. However, 1–2 ml of LIA of similar concentration achieved digital analgesia earlier (<2 min)^24,26^. In lower limb phlebectomy patients, analgesia was achieved within seconds of infiltration of a large volume of 10.0 mg/ml lidocaine and 10.0 µg/ml epinephrine in 1.4 per cent bicarbonate excipient^19^.

Of 10 studies, seven reported the volume of LIA used, excluding the two which used sedative premedications^14,20,21,25,26^. Spearman’s correlation found no significant relation between dose (product of concentration and volume) and latency of lidocaine in all sites (*P* = 0.33), and in fingers specifically (*P* = 0.36). Latency times differed in digits when different volumes of LIA of equal concentrations (10 mg/ml lidocaine and 10 µg/ml epinephrine) were injected (see *Fig. 2*)^20,21,24^. Raised digital compartmental pressures after injecting more than 2 ml of LIA seem to delay analgesia. This reflects the differential analgesic effect of lidocaine; pressure nerve fibres are resistant to lidocaine as compared to pain nerve fibres^11–13^. When using similar volumes, doubling the concentration of lidocaine had no discernible effect on latency^24,26^.

### Latency of epinephrine

Epinephrine’s latency was determined by direct (visualized blood loss) and indirect (non-invasive perfusion monitoring) observations. Direct observation studies (four titles) described stable hypoperfusion after 7.0 min in the eyelid, and after 30.0 min in the upper limb (see *Fig. 3*). Those derived from indirect measurements of perfusion (10 titles) have a pooled mean of 5.6 min (range = 0.5–30.0 min). These include immediate hypoperfusion of finger pulp detected by pulse oximeter (using 6.0 ml of 10.0 mg/ml lidocaine and 20.0 µg/ml epinephrine)^34^, and a maximum of 25.9 ± 5.0 min (using 10.0 ml of 10.0 mg/ml lidocaine and 10.0 µg/ml epinephrine) in the upper arm detected using near-infrared reflectance spectroscopy (NIRS)^35^. The results are summarized below according to anatomic site.

1. In the hand, successful hypoperfusion was achieved by waiting around 30 min^27,30^. The maximum blood loss was approximately double when waiting 7.0 min as compared to 30.0 min^30^. Waiting 5.3 min (3.0–8.0 min) resulted in a 7.7 per cent hypoperfusion failure rate^26^. Waiting only 10.0 min resulted in poor operative field visibility^27^.
2. In the wrist (carpal tunnel surgery), waiting for 33.0 min (23.0–45.0 min) was associated with less bleeding during the first minute after incision as compared to waiting 7.0 min^28^.
3. Volar forearm skin blanching peaked 4.0 min after injection of 0.5 ml of 10.0 mg/ml lidocaine and 10.0 µg/ml epinephrine^36^.
4. Upper eyelid skin achieved hypoperfusion at 7.0 min, with similar overall blood loss to longer waiting periods. One millilitre of 10.0 mg/ml lidocaine with 12.5 µg/ml epinephrine was used. All patients exceeded 50 years of age^29^.

The results of indirect observation studies are interpreted in the discussion section, in the context of current advances in bio-optics, anatomy and skin perfusion dynamics.

### Waiting strategies in practice

There are a few strategies to make effective use of waiting time. During emergency consults, Lalonde injects LIA for early analgesia and hypoperfusion^42^. Waiting time is thus spent completing counselling and documentation. Many practitioners inject patients during turnover, or between marking incisions and scrubbing, introducing a natural delay of around 10.0 min^42–44^. This is sufficient time if a bloodless field is undesirable, as when assessing tissue viability during flap harvest or wound debridement^43^. Patients undergoing wide-awake surgeries may prefer injection and waiting in a less clinical room^23,44^. Here, patients may be injected lying down and then allowed a coffee break or a visit to the bathroom^42^. For optimal turnover and hypoperfusion, hand surgeons schedule multiple patients with simple procedures to arrive 15–30 min prior to the day’s first surgery^42,45^. By the time the third patient receives LIA, the first patient will have achieved stable hypoperfusion. If a case runs long, it is often of no surgical concern if patients receive LIA 50.0 min prior to the surgery^43^. For bloodless septorhinoplasty, Çakir has incorporated multiple delays into his approach, totalling 10.0–15.0 min^46^. He administers the xylometazoline nasal spray 30.0 min preoperatively. This enhances hypoperfusion, allowing him to use more dilute LIA (2.5 µg/ml epinephrine) in septal mucosa, and obviates the need for nasal packing with adrenaline-soaked gauze. He administers LIA slowly, while monitoring haemodynamic parameters and end-tidal CO_2_ for systemic effects of epinephrine. If blood pressure spikes, he takes a break to allow it to settle. He takes breaks between injecting each nostril, and again while the nurse prepares the surgical field (after the injections). He spends his breaks sipping coffee. There is less nasal swelling, the surgery is completed sooner and it is more enjoyable.

In tumescent liposuction, judicious waiting (10.0–60.0 min) is the norm^47–49^. This improves hypoperfusion and hydrodissection, making suction easier^49^. Longer waiting times can be spent in the operating room, relaxing the patient by dimming lights and administering a sedative^50^. Blanching is used to estimate tissue analgesia. At a centre using VASER (Vibration Amplification of Sound Energy at Resonance) liposuction, tumescent is slowly infiltrated (0.05 mg/ml bupivacaine and 0.14 µg/ml epinephrine in buffered 0.9 per cent saline at 100.0 ml/min)^48^. They wait a further 5.0–6.0 min in the neck, and 10.0–15.0 min in the rest of the body, before starting. In the authors’ practice, waiting time for tumescent liposuction is often spent infiltrating other sites. If a single site is to be infiltrated, infiltration can be done before preparing the surgical field. For reduction mammoplasty under general anaesthesia, Hudson injects 500.0 ml of tumescent in each breast (1.2 mg/ml lidocaine and 1.0 µg/ml epinephrine in Ringer’s lactate)^9^. He then spends 15.0 min preparing the surgical field before starting. For wide-awake breast augmentation, Shimizu *et al.* block intercostal nerves (Th3 to Th6) and wait 10.0 min^50^. They then inject 200.0 ml of tumescent in each breast (0.09 mg/ml lidocaine and 18.0 µg/ml epinephrine in 0.9 per cent saline) before commencing with surgery. For breast augmentation under conscious sedation, patient feedback drives a recommendation for waiting 45.0 min after injection of LIA (in each breast 500.0–780.0 ml of 0.5 mg/ml lidocaine and 1.0 µg/ml epinephrine in buffered 0.9 per cent saline)^51^. Creton *et al.* commence lower limb phlebectomy immediately after injecting 20.0–750.0 ml of tumescent (0.28 mg/ml lidocaine and 0.14 µg/ml epinephrine in 1.4 per cent sodium bicarbonate)^19^.

## Discussion

This paper comprehensively reviews evidence on the latency of lidocaine and epinephrine in LIA. This information is important for providers to deliver effective analgesia and achieve a near-bloodless operative field. The ability to provide wide-awake surgery increases patient comfort, improves patient safety and saves valuable resources.

The pooled mean of the latency of lidocaine was 2.6 min (range 0.4–9.0 min). Epinephrine latency varied considerably between studies. Direct observation studies described stable hypoperfusion after 7.0 min in the eyelid, and after around 30.0 min in the upper limb. Most non-invasive imaging studies conflicted with surgical observation, having a pooled mean of 5.6 min (range = 0.5–30 min).

The latency of lidocaine is influenced by acid–base balance, dosage and concentration, skin thickness, nerve diameter, adjuvants, and other factors. Lidocaine, a weak base (pK_a_ = 7.7), accepts protons in acidic pH, becoming more ionized^52^. The ionized molecule cannot penetrate the neurilemma, hence the observation that LIA is less efficacious in inflamed tissues. Inflammatory hyperperfusion and non-compliance of oedematous tissue accentuate this by limiting the amount of LIA remaining in the tissue. To avoid injection pain and improve the efficacy of LIA, some providers inject in the region surrounding the zone of cellulitis^53^. The pH of lidocaine pre-mixed with epinephrine is highly acidic (pH 3.5–5.5)^54^. Using bicarbonate buffer reduces the dose of lidocaine required to achieve analgesia in phlebectomy^19,55^. There were no studies on the effect of buffer on onset time of LIA in the skin. In the field of dentistry, a meta-analysis of pH-adjusted LIA found no change in latency in healthy tissue, but a reduced latency in inflamed tissue by 1.4 min, and a reduced latency of inferior alveolar nerve block by 1.3 min^56^. The effect of buffering on onset of nerve block is more evident in large nerve blocks because it facilitates penetration of thicker perineurium^57,58^. In end-stage renal disease patients receiving brachial plexus blocks, a positive correlation was observed between metabolic acidosis and the latency time of the anaesthetic^59^. Concerns that the time to prepare buffered LIA negates these gains were countered by Burns *et al.*, who demonstrated buffered LIA has a refrigerator-life of at least 2 weeks^60^.

The relationship of dosage and concentration to LIA latency is difficult to study, because of the heterogeneity of injection sites and inconsistency in reporting the volume used. Regional differences in capillary density and infiltrate dispersion make local concentrations difficult to compare. Dose–response is better studied in the context of nerve blocks. Yang *et al*. found no significant relation between volume, concentration and dosage of ropivacaine to latency^61^. Mosaffa *et al.* found reduced latency when using a larger volume of dilute solution (10.0 ml of 13.0 mg/ml lidocaine) as compared to a smaller volume of concentrated solution of equal dose (7.0 ml of 2.0 mg/ml lidocaine)^62^. However, a similar study found no such relation^63^.

Skin thickness and nerve fibre diameter affect lidocaine latency. The scalp requires a larger volume of LIA compared to the face^25^. Here, solution readily disperses and nerve fibres are generally deep to muscles and aponeurosis. Thinner skin (vertex) has a shorter latency compared to thicker skin (remaining scalp). Shorter latency is also seen in skin overlying sharp prominences (supraorbital ridge, helix, inferior border of mandible), or with loose dermal architecture (eyelids and ear lobules)^25^. Lidocaine’s increased latency in the forehead is explained by the deep location of sensory nerves. Na *et al.* similarly observed shorter duration of local anaesthetic in the supraorbital scalp as compared to that of the frontotemporal scalp^64^. Nerve blocks take more time than LIA due to the thicker perineurium of larger nerves^57,58,65^. The reviewed studies suggest digital nerves may have had longer latency time than small branches of volar forearm cutaneous nerves^20,21^. However, this may be related to discomfort from raised digital compartment pressures.

The role of epinephrine in the onset of analgesia is unclear. The pooled mean of the latency of lidocaine with epinephrine exceeded that of plain lidocaine by 1.0 min, although dissimilar concentrations of lidocaine were used. However, two studies on digital block describe reduced latency on addition of epinephrine^14,21^. In buccal infiltration anaesthesia, different concentrations of epinephrine had no significant relation to articaine’s onset^66^. Hyaluronidase is a ‘spreading factor’ that promotes local diffusion and systemic absorption of lidocaine^67^. Commercial preparations are phosphate-buffered. Adjuvant hyaluronidase improves the onset of analgesia and the success rate of ophthalmic and regional nerve blocks^68–71^. An experimental study in human skin comparing lidocaine–hyaluronidase and plain lidocaine found no significant difference in both analgesia onset and duration^67,71,72^. Hyaluronidase’s effect is masked when larger volumes of solution are infiltrated^68,72^. These findings suggest hyaluronidase significantly affects onset when small volumes are used and/or when LIA needs to penetrate multiple fascial layers to reach nerves. It may facilitate intradermal scalp infiltration and enable fewer injections in sensitive sites. To avoid severe allergic reactions, prior intradermal testing is essential. Dexamethasone added to peripheral nerve blocks improves pain but delays onset of sensory and motor blockade^73^. A study of molar extraction patients found age >30 years and smoking more than 10 cigarettes daily were significant independent predictors of delayed onset of analgesia^51^.

Factors affecting latency of epinephrine are best appreciated by reviewing the current anatomy of the vascular and neural network of the skin. From the deep dermal plexus, vessels ascend to supply discrete angiosomes, which overlap without forming an anastomotic network^74,75^. Through these ‘central angiosomal arteries’ the deep dermal plexus may regulate perfusion to the superficial dermis^74^. Thus, no linear relationship can be assumed between blanching, a superficial dermal phenomenon, and the deeper hypoperfusion which is required for surgery. Lidocaine affects cutaneous vasomotor nerves. These are C-type sympathetic nerves (and parasympathetic in the face). They cause vasoconstriction (*α-*2 and *α-*1 adrenergic) and vasodilation (complex cholinergic)^76^.

The face has pronounced capillary density^77^. LIA injected in this region has greater absorption and systemic effects compared to the rest of the body^46,78,79^. In eyelids, epinephrine’s latency is up to 7.0 min^29^. In facelift surgery, one study observed that waiting 15–20 min was insufficient time for adequate hypoperfusion^80^. For bloodless septoplasty, one study recommended waiting 30.0 min after spraying xylometazoline (*α-*1 adrenergic agonist) and a further 10.0–15.0 min after injecting LIA^46^. The latency is at least 25.0 min in the upper limb^24–26^.

Non-invasive perfusion-imaging techniques have many limitations; readers are directed to the excellent review of Cracowski and Roustit^76^. Individual studies are considered in *Table S3*. An important limitation is that laser Doppler and spectroscopic techniques fall short in localizing depth-dependent changes^39,81^. However, photoacoustic imaging has higher spatial resolution; hybrid imaging techniques can compensate for limitations in individual techniques^82^. Secondly, spectroscopy is susceptible to the ‘window effect’ artefact^35,36^. This is a change in skin optical properties following blanching, which allows light to penetrate deeper. The resultant increase in perfusion signals can be falsely interpreted as hyperaemia. This artefact was very clearly observed using laser-speckle contrast imaging (LSCI) and diffuse reflectance spectroscopy (DRS), but not extended-wavelength DRS^36,38–40^. This artefact may be related to tissue vascularity and the probe’s wavelength range^38–40^. It may be less pronounced peripheral to the injection site^83^. Thirdly, only three studies’ instruments had adequate depth and resolution to gauge the deep dermal plexus^35,38,39^. In thick skin, epidermal thickness can reach 1.4 mm and full skin thickness can exceed 5.0 mm. In thin skin, the epidermis is 0.07–0.12 mm thick and full skin thickness is 1.0–2.0 mm (*Table S3* for measurement depths of instruments)^84^. Lastly, eyelid skin may not be a suitable site for testing perfusion imaging, due to the close spacing of the injection sites and the eyelids’ lack of subcutis making injection difficult to standardize^85^. Hult *et al.*’s intraoperative observations provide strong evidence that observations in eyelid skin cannot be generalized to other sites^29^.

Only two perfusion-imaging studies’ results closely reflect direct observations^35,83^. The first study used NIRS^35^. It reported initial hyperperfusion for about 7 min, and stable hypoperfusion at 25.9 ± 5.0 min. The control (plain lidocaine) produced hyperperfusion for about 90 min. The second study used hyperspectral imaging (HSI)^83^. It used articaine, not lidocaine, but remains relevant to this review. After injecting plain articaine, plain epinephrine, and articaine–epinephrine, they observed injection sites of 6.7 mm radius and peri-injection sites. All sites had initial hyperperfusion. A ring of hypoperfusion started from the periphery of the injection and spread inwards and outwards. In peri-injection sites, stable hypoperfusion was observed after 15.0 min in the plain epinephrine site, and after 30.0 min in articaine–epinephrine sites. Sites injected with epinephrine or articaine–epinephrine showed persistent hypoperfusion throughout the observation period (120.0 min). Sites injected with plain articaine showed a persistent hyperperfusion signal.

Building on others’ hypotheses, the following inferences were drawn from perfusion-imaging studies.

1. Injection causes a transient hyperperfusion due to a histaminic response. Injecting 0.9 per cent saline led to increased flow on laser Doppler imaging (LDI), lasting 7.0 min in the forearm and 2.0 min in the face^33^. Variation may reflect different concentrations of dermal mast cells^86^. This response is less pronounced peripheral to the injection site^84^.
2. Hyperperfusion after injecting epinephrine suggests a ‘window effect’ artefact. Blanching results in measurement of the deeper plexuses, which are better perfused. This hyperperfusion was transient in the two studies, strongly indicating that the deep dermal plexus or the subcutaneous plexus was observed^35,84^.
3. Deeper tissues may have a longer latency period than superficial tissues (when using the standard concentration of epinephrine in LIA). Stimulation of α-2 adrenoceptors induces a redistribution of blood flow from the superficial dermis to the deeper plexus^77^. This is a thermoregulatory response to cold. In the superficial dermis, stable hypoperfusion was seen about 2 min after injecting LIA^39,40^. Blanching, a superficial dermal phenomenon, peaks about 4 min after epinephrine injection^36^. This review found four studies on lidocaine with epinephrine LIA with instruments of sufficient penetration depth to observe the deep dermis. Only one was consistent with surgical observations (latency of 25.9 ± 5.0 min)^35^. This study was subject to the ‘window effect’ artefact. Of the remaining studies, one used intradermal injection^39^, one focused on arterial saturation^37^, and one showed paradoxical hypoperfusion after 0.9 per cent saline injection and thus could not be relied upon^38^.
4. Subcutaneously injected epinephrine is unlikely to travel vertically to constrict superficial dermal vessels. Travel between planes is very slow; even after 10.0 min, intradermal injection showed no effect on the deep dermal plexus^39^. This is because a uniform collagen-dense layer of reticular dermis divides these regions^87^. This phenomenon is readily seen during scalp surgery; injection into either the dermis or subdermis does not readily produce hypoperfusion in the adjacent plane.
5. The above points support the hypothesis that the deep dermal plexus may contain a regulatory mechanism that acts on the ‘central angiosomal arteries’ arising from it, thus modulating perfusion to superficial dermis^75^.

There are a few hypotheses to explain epinephrine’s latency. Epinephrine must overcome the vasodilatory effects of lidocaine and histamine^28,35^. Time may be spent in diffusion to deeper tissues to constrict larger feeding vessels^28^. The authors propose that the deep dermal plexus is less sensitive to epinephrine compared to its superficial branches.

This systematic review focuses on cutaneous anaesthesia. It is strong for concisely reviewing an expansive and relevant topic, for reviewing quality studies, and for providing insight regarding many conflicting observations by considering current literature on microanatomy, pharmacodynamics and perfusion imaging. The waiting strategies discussed reveal the diversity in expert opinions and can inform practice.

It is limited by variations in studies’ anatomic sites, concentrations of LIA solution and non-invasive imaging modalities. Many studies observe non-healthy participants. There are very few studies that quantify intraoperative bleeding after epinephrine injection, with variable waiting times. Multimodal analysis and hybrid imaging techniques that target the deep dermal plexus can better describe skin perfusion dynamics in response to epinephrine injection. Perfusion studies such as NIRS and HSI are promising as they have been shown to correlate clinically with blood loss^35,83^.

## Supplementary Material

zrad089_Supplementary_Data

## Data Availability

Data supporting the findings of this study are available within the article and its supplementary materials. Further information will be made available upon reasonable request.
