# Supplementary material for: How long to wait after local infiltration anaesthesia: systematic review
Source: BJS Open. 2023 Sep 28;7(5):zrad089. doi: 10.1093/bjsopen/zrad089 (PMC10538258; doi:10.1093/bjsopen/zrad089)
Supplement: zrad089_Supplementary_Data [file zrad089_supplementary_data.docx]

**Title:** How long to wait after local infiltration anaesthesia: systematic review

**Authors:**

Mohammad Suleman Bajwa, MBBS^1^

Muhammad Mustehsan Bashir, MBBS, FCPS, MME, PhD^1^

Mohammad Hamza Bajwa, MBBS^2^

Zafar Iqbal, MBBS^1^

Muhammad Aizaz Salahuddin^1^

Ahmad Hussain Hocane^1^

Farooq Shahzad, MBBS, MS, FACS, FAAP^3^

**Author Affiliations:**

^1^ Department of Plastic & Reconstructive Surgery/Mayo Burn Centre, Mayo Hospital, King Edward Medical University, Lahore, Pakistan

^2^ Department of Neurosurgery, Aga Khan University, Karachi, Pakistan

^3^ Plastic & Reconstructive Surgery Service, Memorial Sloan-Kettering Cancer Center, New York, USA

**Corresponding Author:**

Mohammad Suleman Bajwa, MBBS

Department of Plastic & Reconstructive Surgery/Mayo Burn Centre, Surgical Tower, Mayo Hospital, Lahore, Hospital Rd, Anarkali Bazaar, Lahore, Punjab 54000, Pakistan

Email: suleman97@outlook.com

Tel: +92-3014237057

ORCiD: 0000-0002-8485-4366

**Supplementary Materials - Index**

| **Supplementary Tables** |  |
| --- | --- |
| Table 1: Characteristics of studies on onset of analgesic effect | *pag. 3* |
| Table 2: Characteristics of studies on onset of hypo-perfusion - measured directly by peroperative bleeding | *pag. 5* |
| Table 3: Characteristics of studies on onset of hypo-perfusion - measured indirectly by non-invasive perfusion imaging techniques | *pag. 6* |
| **References** | *pag. 8* |
|  |  |

**Supplementary Tables**

**Table 1: Characteristics of studies on onset of analgesic effect**

|  | Article | Population | Age range (years) | Means of outcome measurement | Outcome  (min) | Local anesthetic concentration | | Volume  (ml) | Anatomic site | Control group(s)* | Comments |
| --- | --- | --- | --- | --- | --- | --- | --- | --- | --- | --- | --- |
|  |  |  |  |  |  | Lido (mg/ml) | Epi (μg/ml) |  |  |  |  |
| 1 | Maimon  et al  (1984)^13^ | 28 patients (13 received lido-epi) (facial surgery) | 16**-**64 | pain to pinprick | 2.1 (1**-**4) | 10 | 5 | **-** | face | no control | One patient had pain on incision (required repeat injection)  Preop. sedation was used |
|  |  |  |  | pain on incision | 3.5 (2**-**8) |  |  |  |  |  |  |
| 2 | Alhelali  et al (2009)^20^ | 12 healthy volunteers | 27 ± 4 | pain to pinprick | 3.5 (3**-**8) | 10 | 10 | 4 | middle finger base (ring block) | no control |  |
| 3 | Sonohata  et al (2012)^21^ | 9 healthy volunteers | 20**-**37 | pain to pinprick (self**-**induced) | 2.8 ± 0.8 | 10 | 10 | 3 | finger base | 10mg/ml lido | Latency of lido 4 ± 0.9 min |
| 4 | Creton  et al (2012)^19^ | 215 patients (varicose vein surgery) | 19**-**91 | pain on incision | <1 min | 0.28 | 0.14 | 20**-**750 | lower limb | no control | Some patients received premedicated w/ bromazepam or homeopathic granules |
| 5 | Collins  et al (2013)^14^ | 25 healthy volunteers | 36 ± 9.7 | pain to pinprick | 0.4 ± 0.8 | 10 | 10 | 0.2 | volar forearm | 10mg/ml lido | Latency of lido 0.5min  Injections administered intradermally |
| 6 | Prasetyono  et al (2016)^22^ | 12 healthy male volunteers | 18**-**30 | pain to SW monofilament and 2**-**point discrimination | 5 (1**-**9) | 2 | 1 | till pale | finger pulp | 20 mg/ml lido | Latency of lido was 1(1**-**6) min  (Note: lido affects pain more than mechanoreception) |
| 7 | Öztürk et al (2018)^23^ | 52 patients (hand surgery) | 18**-**64 | pain on incision | 5.3 (3**-**8) | 10 | 10 | **-** | hand | axillary block (n=26) | Bleed requiring tourniquet (n=2) minimal bleed (n=10), no bleed (n=14) |
| 8 | Rashid et al (2019)^24^ | 86 patients (hand surgery) (43 received lido-epi) | 35-85 | pain on 26-gauge needle-prick | 1.4 | 10 | 10 | 1 ml | trigger finger base | 10mg/ml lido | Latency of lido = 1.7 (*P* = 0.2)  After recording pain score a further 3 ml of lido-epi was administered |
| 9 | Safran et al (2019)^25^ | 102 patients (surgical excision of head & neck cancer 72.5% BCC, 26.5% SCC) | 74.3 (mean) | pain to pinprick (center and surroundings of lesion) | 2.6 | 10 | 10 | 6.0 | upper face  (n=30) | no control | The ‘upper face’ (forehead and scalp above level of lateral canthus) look more time than other regions (*P<0.01* by t test)  Shortest time: eyebrow (0.5 min, n=1), longest time: lower forehead (4.18, n=7)  One injection was performed per patient, with the largest volume used in occipital scalp: 11.5 ml (n=2), and least volume used in helix: 1.3 ml (n=2) |
|  |  |  |  |  | 1.2 | 10 | 10 | 4.7 | lower face  (n=62) |  |  |
|  |  |  |  |  | 1.0 | 10 | 10 | 3.7 | ear  (n=10) |  |  |
| 10 | Córdoba**-**Fernández et al (2019)^26^ | 112 healthy volunteers (28 received lido-epi) | 24 ± 7 | pain to pinprick | 1.6 ± 0.4 | 20 | 10 | 2 | 2nd toe | 20mg/ml lido | Latency of lido 2.3 ± 0.7 min *(P<0.01)* |

*lido = lidocaine, epi = epinephrine, SW monofilament = Semmes-Weinstein monofilament, BCC = basal cell carcinoma, SCC = squamous cell carcinoma*

**groups containing non-lidocaine local anesthetics (with or without epinephrine) are not included in table*

**Table 2: Characteristics of studies on onset of hypo-perfusion - measured directly by peroperative bleeding**

|  | Article | Population | Age range (years) | Means of outcome measurement | Outcome  (min) | Local anesthetic concentration | | Volume  (ml) | Anatomic site | All groups | Comments |
| --- | --- | --- | --- | --- | --- | --- | --- | --- | --- | --- | --- |
|  |  |  |  |  |  | Lido  (mg/ml) | Epi  (μg/ml) |  |  |  |  |
| 1 | Bashir et al (2015)^27^ | 75 patients (elective & emergency hand surgery) | 16**-**60 | bleeding estimated by operative field visibility | ⁓25 | 1.80 | 4.52 | till firm and pale | flexor surface of hand | 10, 15, 25 min wait | Rescue tourniquet needed in all 10 min cases on interim analysis |
| 2 | Mckee et al (2015)^28^ | 15 patients (carpal tunnel decompression surgery) | 54 (mean) | bleeding measured by micropippette (in first minute of incision) | 33 (23**-**45) | 10 | 10 | 10 | over carpal tunnel | 7, 33 min wait | Blood loss 0.17 ± 0.08 ml/cm (7min group) and 0.06 ± 0.03 ml/cm (33min group) *(P<0.05)*  7 min wait patients had per**-**op neuralgia unlike the 33 min wait group which had none |
| 3 | Hult et al (2018)^29^ | 16 patients (blepharoplasty) | 51**-**78 | bleeding measured by surgical swab weight (swabbing at 5 min intervals) | 7 | 10 | 12.5 | 1 | eyelid | 7, 15 and 30 min wait for lido**-**epi,  1 min wait for control (20 mg/ml lido) | Blood loss was 75% less in experimental group compared to control  Blood loss comparable in all groups – waiting 7 min was found as effective as longer waiting times |
|  |  |  |  |  |  |  |  |  |  |  |  |
| 4 | Hernandez et al (2020)^30^ | 34 patients (hand surgery) | 21**-**75 | bleeding estimated by gauze visual analogue scale | 30 | 10 | 10 | **-** | hand | 7, 30 min wait | Blood loss 8 ± 5.8 ml (7 min group) and 5 ± 2.2 ml (30 min group) *(P=0.07)*, complication rates 18% (7 min group) and 0% (30 min group) *(P=0.22)*, similar pain scores and operative times (6**-**12 min) |
|  |  |  |  |  |  |  |  |  |  |  |  |
|  |  |  |  |  |  |  |  |  |  |  |  |

*lido = lidocaine, epi = epinephrine*

**Table 3: Characteristics of studies on onset of hypo-perfusion - measured indirectly by non-invasive perfusion imaging techniques**

|  | Article | Population | Age range (years) | Means of outcome measurement | Outcome  (min) | Local anesthetic concentration | | Volume  (ml) | Anatomic site | Control group(s)* | Comments |
| --- | --- | --- | --- | --- | --- | --- | --- | --- | --- | --- | --- |
|  |  |  |  |  |  | Lido  (mg/ml) | Epi  (μg/ml) |  |  |  |  |
| 1 | O'Malley et al (1995)^31^ | 23 patients (elective head & neck surgery) | **-** | LDI (633 nm, 0.5mm depth) | 3**-**4 min  (for each conc. of epi) | 10 | 2.5, 5, 10, 20 | 3 | head & neck | 0.9% saline, 10 mg/ml lido | 0.9% saline and lido remained above baseline (observed for 10 min)  Under general anesthesia |
| 2 | Dunlevy et al (1996)^32^ | 81 patients (elective head & neck surgery) | **-** | LDI (633 nm, 0.5mm depth) | 4**-**5 min  (for each conc. of epi) | 10 | 1.25, 2.5, 5, 10 | 3 | head & neck | 10 mg/ml lido | Lido returned to baseline flow within 10 min  Under general anesthesia |
| 3 | Ghali et al (2008)^33^ | 5 healthy volunteers | 25**-**40 | LDI (633 nm, 0.5mm depth) | 10 | 10 | 10 | 0.5 | volar forearm | phosphate buffered 0.9% saline, 10 mg/ml lido | 4 °C solution injected (for standard temperature) |
|  |  |  |  |  |  |  |  |  |  |  |  |
|  |  | 5 healthy volunteers | 25**-**35 | LDI (633 nm, 0.5mm depth) | 8  (for each conc. of epi) | 10 | 5, 10 | 0.5 | face | phosphate buffered 0.9% saline, 10 mg/ml lido |  |
|  |  |  |  |  |  |  |  |  |  |  |  |
| 4 | Hafner et al (2008)^34^ | 20 healthy volunteers | 19**-**27 | LDF (780 nm, 0.5mm depth) | <1 min | 10 | 5 | 6 | finger (Oberst’s block) | 0.9% saline, lido | Hypoperfusion in 0.9% saline group (vasocompression by hydropressure) |
| 5 | Mckee et al (2013)^35^ | 12 healthy volunteers | 21**-**31 | NIRS (700**-**2500nm, ~10mm depth) (focused on wavelengths of Dawson Hb index) | 25.9 ± 5min | 10 | 10 | 5 | lateral arm | 10 mg/ml lido | Initial hyperperfusion lasting ⁓80 min in control group (lido), and ⁓7 min in the experiment group (lido-epi). |
|  |  |  |  |  |  |  |  |  |  |  |  |
| 6 | Sheikh et al (2019)^36^ | 12 healthy volunteers  (78% Fitzpatrick 2) | 31**-**72 | RGB analysis of blanching (0.35**-**0.45mm depth) | 9 (4**-**16) | 20 | 12.5 | 0.5 | volar forearm | 20 mg/ml lido | Maximum blanching was observed after ⁓4 min (stable τ is mentioned left)  ‘window effect’ observed with LSCI |
|  |  |  |  |  | 7 (4**-**12) | 10 | 20 | 0.5 |  |  |  |
|  |  |  |  | LSCI (785nm, 0.7mm depth) | **-** |  |  |  |  |  |  |
|  |  |  |  |  |  |  |  |  |  |  |  |
| 7 | Prasetyono et al (2019)^37^ | 12 healthy volunteers  (male) | 21**-**30 | PO (660**-**940 nm, 5mm depth) | 13.9 ± 5.4 (range = 2**-**20.8) | 2 | 1 | **-** | finger pulp | 0.9% saline | EMLA was used in both groups,  PO detects arterial sO_2_ only |
| 8 | Bunke et al (2019)^38^ | 9 healthy volunteers | 24**-**69 | Extended**-**wavelength DRS (450**-**1550nm, 1.25mm depth) (focused on wavelengths of Dawson Hb index) | 2.6 | 20 | 12.5 | 0.5 | volar forearm | 0.9% saline | No ‘window effect’  Pulse oximetry readings were ≥97% in all cases |
|  |  |  |  | PO (660**-**940 nm, 5mm depth) | **-** | **-** | **-** | **-** | **-** |  |  |
| 9 | Bunke et al (2021)^39^ | 7 healthy volunteers | 20**-**75 | DRS (500**-**650nm, ~1mm depth) | 1.8 | 20 | 12.5 | 0.5 | volar forearm | no control | Injection was administered intra**-**dermally, hbo_2_ assessed in same region |
|  |  |  |  |  |  |  |  |  |  |  |  |
|  |  |  |  | Photoacoustic imaging (680**-**970nm, 20mm depth set to 5 mm) | 2.1 |  |  |  |  |  | Hypodermis was not affected in the observation period (6**-**10 min) |
|  |  |  |  |  |  |  |  |  |  |  |  |
|  |  |  |  |  |  |  |  |  |  |  | After 40 sec, DRS showed paradoxical results (‘window effect’) |
| 10 | Bunke et al (2022)^40^ | 9 patients (blepharoplasty) (100% Fitzpatrick 2) | 46**-**74 | LSCI for perfusion (785nm, 0.7mm depth) | 1.9 | 20 | 12.5 | 1 | eyelid | no control | (t^1/2^ is time to half maximal effect and is ⁓1.5min at point of injection, and ⁓4min at 4mm from point of injection)  High vascularity of the eyelid may have mitigated the ‘window effect’ |
|  |  |  |  | HSI for sO_2_ (500**-**1000 nm, ~1mm depth) | **-** |  |  |  |  |  |  |

*lido = lidocaine, epi = epinephrine, conc. = concentration, DRS = diffuse reflectance spectroscopy, HSI = hyperspectral imaging, LDI = laser Doppler imaging, LDF = single****-****point laser Doppler imaging, LSCI = laser****-****speckle contrast imaging, NIRS = near infrared tissue reflectance spectroscopy, PO = pulse oximetry, RGB = red****-****green****-****blue camera, sO_2_ = tissue/blood oxygen saturation (=HbO_2_/Hbt)*

**groups containing non-lidocaine local anesthetics (with or without epinephrine) are not included in table*

**References**

**The references are also used in the manuscript and are numbered accordingly.**

| 13 | Maimon WN, Schuller DE. Lidocaine v bupivacaine in facial plastic surgery: A clinical trial. Archives of Otolaryngology. 1984 Aug 1;110(8):525-8. |
| --- | --- |
| 14 | Collins JB, Song J, Mahabir RC. Onset and duration of intradermal mixtures of bupivacaine and lidocaine with epinephrine. Canadian Journal of Plastic Surgery. 2013 Mar;21(1):51-3. |
| 19 | Creton D, Rea B, Pittaluga P, Chastanet S, Allaert FA. Evaluation of the pain in varicose vein surgery under tumescent local anaesthesia using sodium bicarbonate as excipient without any intravenous sedation. Phlebology. 2012 Oct;27(7):368-73. |
| 20 | Alhelail M, Al-Salamah M, Al-Mulhim M, Al-Hamid S. Comparison of bupivacaine and lidocaine with epinephrine for digital nerve blocks. Emergency Medicine Journal. 2009 May 1;26(5):347-50. |
| 21 | Sonohata M, Nagamine S, Maeda K, Ogawa K, Ishii H, Tsunoda K, Asami A, Mawatari M. Subcutaneous single injection digital block with epinephrine. Anesthesiology Research and Practice. 2012 Jan 1;2012. |
| 22 | Prasetyono TO, Lestari PA. The onset and duration of action of 0.2% lidocaine in a one-per-mil tumescent solution for hand surgery. Archives of plastic surgery. 2016 May;43(03):272-7. |
| 23 | Öztürk İA, Orman O, Baydar M, Aykut S, Köse A. Comparison of the Cost and Efficacy of Axillary Anesthesia and Wide-Awake Anesthesia in Finger Surgeries. The Medical Bulletin of Sisli Etfal Hospital. 2018;52(2):119. |
| 24 | Rashid MZM, Sapuan J, Abdullah S. A randomized controlled trial of trigger finger release under digital anesthesia with (WALANT) and without adrenaline. Journal of Orthopaedic Surgery. 2019 Mar 8;27(1):2309499019833002. |
| 25 | Safran T, Zammit D, Kanevsky J, Khanna M. Efficacy of Local Anesthesia in the Face and Scalp: A Prospective Trial. Plastic and Reconstructive Surgery Global Open. 2019 May;7(5). |
| 26 | Córdoba-Fernández A, González-Benítez J, Lobo-Martín A. Onset time of local anesthesia after single injection in toe nerve blocks: a randomized double-blind trial. Journal of Perianesthesia Nursing. 2019 Aug 1;34(4):820-8. |
| 27 | Bashir MM, Qayyum R, Saleem MH, Siddique K, Khan FA. Effect of time interval between tumescent local anesthesia infiltration and start of surgery on operative field visibility in hand surgery without tourniquet. The Journal of Hand Surgery. 2015 Aug 1;40(8):1606-9. |
| 28 | McKee DE, Lalonde DH, Thoma A, Dickson L. Achieving the optimal epinephrine effect in wide awake hand surgery using local anesthesia without a tourniquet. Hand. 2015 Dec;10(4):613-5. |
| 29 | Hult J, Sheikh R, Nguyen CD, Tenland K, Dahlstrand U, Malmsjö M. A waiting time of 7 min is sufficient to reduce bleeding in oculoplastic surgery following the administration of epinephrine together with local anaesthesia. Acta Ophthalmologica. 2018 Aug;96(5):499-502. |
| 30 | Hernandez A, Rosario M, Mendoza-Torres R, Taguba CR, Garcia A, Battad G. Evaluating clinical outcomes for determining the optimal delay to skin incision under WALANT: a prospective series of 34 patients from a low-resource tertiary setting. Advances in orthopedics. 2020 Aug 15;2020. |
| 31 | O'Malley TP, Postma GN, Holtel M, Girod DA. Effect of local epinephrine on cutaneous blood flow in the human neck. The Laryngoscope. 1995 Feb;105(2):140-3. |
| 32 | Dunlevy TM, O'Malley TP, Postma GN. Optimal concentration of epinephrine for vasoconstriction in neck surgery. The Laryngoscope. 1996 Nov;106(11):1412-4. |
| 33 | Ghali S, Knox KR, Verbesey J, Scarpidis U, Izadi K, Ganchi PA. Effects of lidocaine and epinephrine on cutaneous blood flow. Journal of plastic, reconstructive & aesthetic surgery. 2008 Oct 1;61(10):1226-31. |
| 34 | Häfner, Hans-Martin, . "Changes in acral blood flux under local application of ropivacaine and lidocaine with and without an adrenaline additive: a double-blind, randomized, placebo-controlled study." Clinical hemorheology and microcirculation 38.4 (2008): 279-288. |
| 35 | McKee DE, Lalonde DH, Thoma A, Glennie DL, Hayward JE. Optimal time delay between epinephrine injection and incision to minimize bleeding. Plastic and Reconstructive Surgery. 2013 Apr 1;131(4):811-4. |
| 36 | Sheikh R, Bunke J, Thorisdottir RL, Hult J, Tenland K, Gesslein B, Reistad N, Malmsjö M. Hypo-perfusion following the injection of epinephrine in human forearm skin can be measured by RGB analysis but not with laser speckle contrast imaging. Microvascular Research. 2019 Jan 1;121:7-13. |
| 37 | Prasetyono TO, Kusumastuti N. Optimal time delay of epinephrine in one-per-mil solution to visualize operation field. Journal of Surgical Research. 2019 Apr 1;236:166-71. |
| 38 | Bunke J, Sheikh R, Reistad N, Malmsjö M. Extended-wavelength diffuse reflectance spectroscopy for a comprehensive view of blood perfusion and tissue response in human forearm skin. Microvascular Research. 2019 Jul 1;124:1-5. |
| 39 | Bunke J, Merdasa A, Sheikh R, Albinsson J, Erlöv T, Gesslein B, Cinthio M, Reistad N, Malmsjö M. Photoacoustic imaging for the monitoring of local changes in oxygen saturation following an adrenaline injection in human forearm skin. Biomedical Optics Express. 2021 Jul 1;12(7):4084-96. |
| 40 | Bunke J, Merdasa A, Stridh M, Rosenquist P, Berggren J, Hernandez-Palacios JE, Dahlstrand U, Reistad N, Sheikh R, Malmsjö M. Hyperspectral and Laser Speckle Contrast Imaging for Monitoring the Effect of Epinephrine in Local Anesthetics in Oculoplastic Surgery. Ophthalmic Plastic & Reconstructive Surgery. 2022 Apr 25:10-97. |
